# Intracellular nonequilibrium fluctuating stresses indicate how nonlinear cellular mechanical properties adapt to microenvironmental rigidity

**DOI:** 10.1038/s41598-020-62567-x

**Published:** 2020-04-03

**Authors:** Ming-Tzo Wei, Sabrina S. Jedlicka, H. Daniel Ou-Yang

**Affiliations:** 1 Department of Bioengineering, Bethlehem, Pennsylvania 18015 USA; 2 Department of Materials Science and Engineering, Bethlehem, Pennsylvania 18015 USA; 3 Department of Physics, Lehigh University, Bethlehem, Pennsylvania 18015 USA

**Keywords:** Biophysics, Soft materials, Biological physics, Thermodynamics

## Abstract

Living cells are known to be in thermodynamically nonequilibrium, which is largely brought about by intracellular molecular motors. The motors consume chemical energies to generate stresses and reorganize the cytoskeleton for the cell to move and divide. However, since there has been a lack of direct measurements characterizing intracellular stresses, questions remained unanswered on the intricacies of how cells use such stresses to regulate their internal mechanical integrity in different microenvironments. This report describes a new experimental approach by which we reveal an environmental rigidity-dependent intracellular stiffness that increases with intracellular stress - a revelation obtained, surprisingly, from a correlation between the fluctuations in cellular stiffness and that of intracellular stresses. More surprisingly, by varying two distinct parameters, environmental rigidity and motor protein activities, we observe that the stiffness-stress relationship follows the same curve. This finding provides some insight into the intricacies by suggesting that cells can regulate their responses to their mechanical microenvironment by adjusting their intracellular stress.

## Introduction

The mechanical integrity of cells is maintained by molecular motors in cytoskeleton networks, which form coupled dynamic mechanical systems. Dynamic mechanical interactions affect functions such as cell spreading and stiffness^1–4^ as well as stem-cell differentiation^5,6^. It has been suggested that these responses can be regulated by traction stress arising from molecular motors^6–8^. To investigate how the mechanical properties of cytoskeleton networks respond to stress, prior studies demonstrated a stress-dependent stiffness in a synthesized cytoskeletal network *in vitro*^9–13^. Such a nonlinear mechanical response was also found in the elastic response of biological cells to extracellular stress^14–18^. It has been hypothesized that a cell can generate intracellular contractile stress to help them sense and adapt to their microenvironments. However, the relation between intracellular stress and stiffness to underlying microenvironmental rigidity has yet to be described.

**Figure 1:**
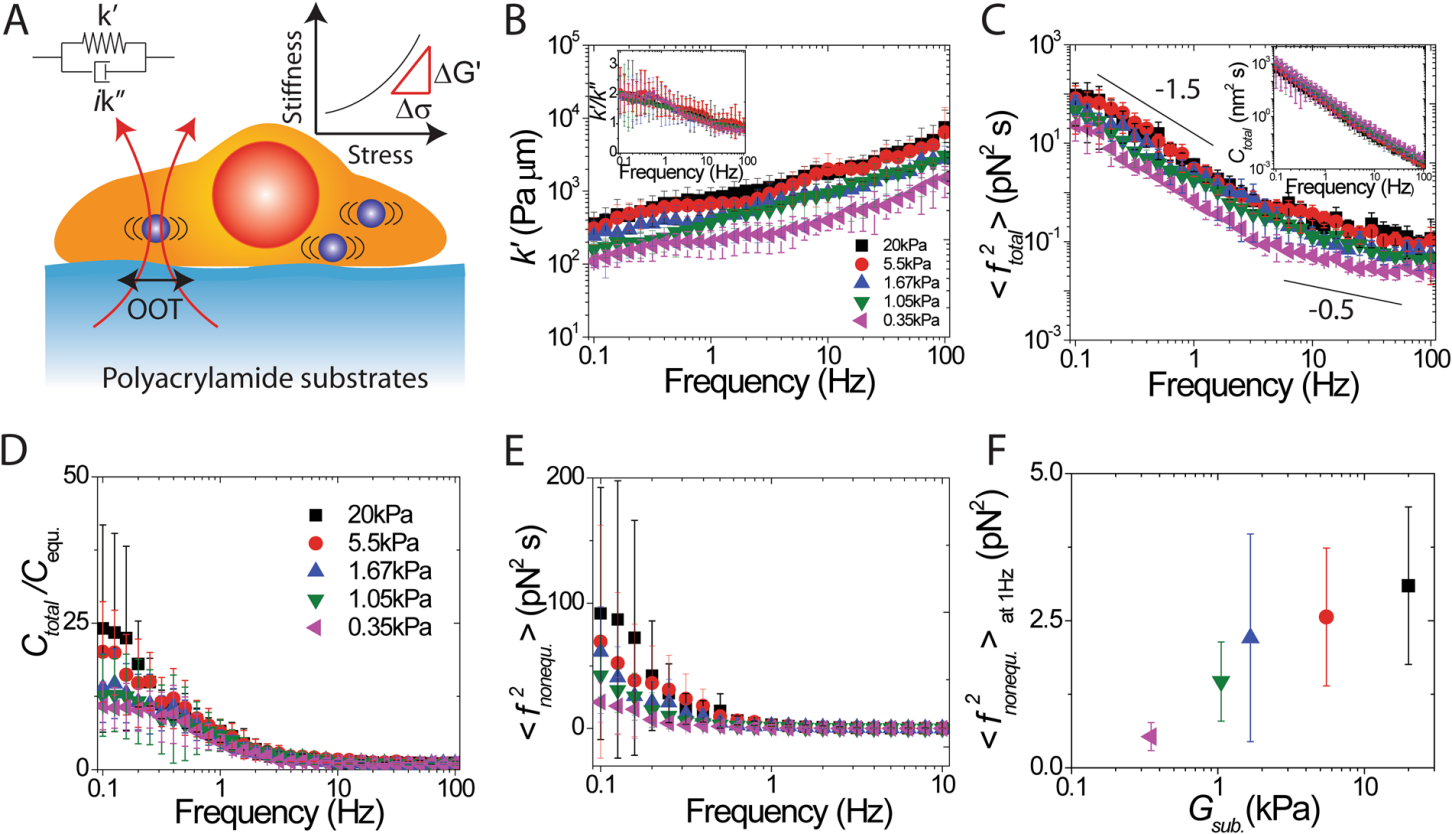
Measurements of intracellular fluctuating force spectrum using a combination of active and passive microrheology. (**A**) Sketch of the two intracellular microrheology techniques. In active microrheology, the effective complex spring constant k^*^ *=* *k*′ + *ik*″ is measured directly by an oscillatory optical tweezers (OOT) acting on a trapped intracellular probe particle. In passive microrheology, a CCD camera tracks fluctuation in the probe-particle position. (**B**) Intracellular stiffness *k*′ for cells cultured on substrates with elastic moduli varying from 0.35 kPa to 20 kPa. *k*′ follows a weak power-law dependence on frequency. Insert shows a decreasing solid-like behavior *k*′*/k*″ (ratio of stiffness to dissipative resistance) as a function of frequency. (**C**) The total intracellular fluctuating force spectra $\langle \,{f}_{total}^{2}\rangle$ increase with substrate rigidity. Insert shows a total fluctuation spectrum as a function of frequency. (**D**) *C*_*total*_ */C*_*equ*._ as a function of frequency varies with the substrate rigidity. (**E**) Nonequilibrium fluctuating force spectra $\langle \,{f}_{nonequ.}^{2}\rangle$ as a function of frequency varies with the substrate rigidity (**F**) Nonequilibrium fluctuating force at 1 Hz increases with substrate rigidity. Colors indicate elastic moduli of the cell-culture substrates (see Fig. 1D legend). *G*_*sub*._ is the elastic modulus of the substrate. Error bars are standard deviation of the mean from ten independent measurements from individual probed particles in each cell.

To elucidate how intracellular stresses affect intracellular mechanical properties, we use force spectrum microscopy^19–21^ (Fig. 1A) to study the intracellular local stress. In this paper, we examine how to use the ratio of the fluctuations in the intracellular stiffness modulus to the intracellular nonequilibrium fluctuating stress to determine the stress level; the slope of the stress-dependent stiffness as shown in Fig. 1A. Here, combining passive and active microrheology and using an internalized micron-sized particle as a probe, we are able to distinguish the power spectrum of nonequilibrium fluctuating forces from that of a thermal fluctuating force. Moreover, the ratio of the fluctuations in the intracellular stiffness modulus to the intracellular nonequilibrium fluctuating stress is never zero. The ratio increases with increasing intracellular time-averaged stiffness modulus, indicating nonlinear mechanical behavior. Taking this ratio as a derivative, we integrate it as a function of cell stiffness modulus to obtain the intracellular stress as a function of cell stiffness modulus. This information has been difficult to obtain in previous approaches^6,7,15,22^. We also demonstrate that intracellular stiffness modulus as a function of intracellular stress obeys a nonlinear curve with power-law dependence by treating intracellular motor proteins with inhibitory drugs. This result suggests that cells can regulate their mechanical properties by adjusting their intracellular stress. The data reveal the relationship between molecular dynamics and emergent mesoscale material properties in living cells, thus inspiring further research on how living systems can take advantage of fluctuations in nonlinear systems.

## Results

### Intracellular fluctuating forces increase with substrate rigidity

To examine how cells adjust their intracellular mechanical properties to adapt their microenvironment, we use active microrheology (AMR) approach^12,21–24^ by using optical tweezers to trap and oscillate harmonically a 1 μm engulfed polystyrene particle inside a living HeLa cell. From the displacements during the particle oscillation, cellular internal responses to the applied forces are determined. Internal responses are captured in the effective spring constant of the following linear relation: *F *= k^*^u where *F* is the force acting on the particle, *u* is the position of the particle and k^*^ is a complex effective spring constant. The real and imaginary parts of the complex effective spring indicate an stiffness, *k*′ and dissipative resistance, *k*″, respectively. Both *k*′ and *k*″ are determined from the experimentally measured displacement magnitude and phase of the particle^21,24^. We find that cells attached to a stiffer substrate (20 kPa polyacrylamide gel, coated with collagen) are significantly stiffer than those attached to a more compliant substrate (0.35 kPa polyacrylamide gel, coated with collagen) as shown in Fig. 1B. These results are qualitatively consistent with previous extracellular measurements using atomic force microscopy to determine whole cell stiffness to response substrate rigidity^25^. Additionally, the intracellular stiffnesses follow a power-law dependence on frequency, similar to those of a soft glass^22,24^. In the frequency range 0.1 to 10 Hz, the HeLa cells exhibit a relatively solid-like response (i.e., *k*′/ *k*″ > 1, shown in the insert of Fig. 1B), similar to the cellular cortex^26^ and the synthesized active cytoskeletal networks^12^ measurements. Notably, the solid-like response depends on frequency, which is consistent with the typical release rate of myosin filaments observed in synthesized cytoskeletal networks^12^. However, it is surprising that this solid-like response weakly depends on the microenvironmental rigidity, which indicates both that the viscoelastic properties are adjusted through intracellular force.

To determine how intracellular force responds to substrate rigidity, we combine both active and passive cellular microrheology approaches. The passive method^19,20^ is based on a statistical analysis of thermal fluctuations of internalized particle motion. Here, the total fluctuating force spectrum^20^ $\langle {f}_{total}^{2}\rangle$ can be determined as $\langle {f}_{total}^{2}\rangle ={\langle {k}^{\ast }\rangle }^{2}{C}_{total}$, where k^*^ is measured by AMR and *C*_*total*_ is the total fluctuation of the probe particle position (the insert of Fig. 1C) measured by passive microrheology (PMR). We refer to fluctuations measured by PMR as **“**total” fluctuations, since they represent the response of the probe particle to an intracellular medium that contains both passive thermal-equilibrium and active forces (see below). The total fluctuating force as a function of frequency follows a power law with exponent about −1.5 at the lower (0.1~10 Hz) frequencies and about −0.5 at the higher frequencies (10~100 Hz)^27^ as shown in Fig. 1C. The power-law behavior implies that the microscopic processes responsible for active stress have a broad distribution of activation rates^28^. Compared with previous reports showing $\langle {f}_{total}^{2}\rangle  \sim {\omega }^{-2}$ ^28–30^, our low-frequency exponent of −1.5 indicates that intracellular motors^29^ are less abundant and/or less active than motors on the cellular cortex^28,30^, implying that the locally intracellular tension is smaller than tension on the cellular cortex. The behaviors of total fluctuating force spectra indicate that the thermal fluctuation effect would be comparable with the nonequilibrium contribution from intracellular forces inside a living cell.

To address the nonequilibrium contribution, we combine AMR and PMR to distinguish the power spectrum of nonequilibrium forces from that of a thermal force^28,29,31^. Here, the fluctuations of a probe particle measured by PMR show “total” fluctuations including both equilibrium and nonequilibrium fluctuations. The thermal-equilibrium fluctuation spectrum (*C*_*equ*._) of a probe particle can be found using AMR measurements of the imaginary part *k*″ of the effective spring constant k*^24^. In an equilibrium system, only thermal-equilibrium forces act on the probe, and the power spectral density of the displacement fluctuations is related to the mechanical response of the material by the fluctuation-dissipation theorem *C*_*equ*._ = *2k*_*B*_*T/k*″*ω*, where *k*_*B*_ is Boltzmann’s constant and *T* is the absolute temperature. Fig. 1D shows the ratio of the total-fluctuation power spectrum, measured by passive microrheology, to that of equilibrium fluctuations estimated by active microrheology. This ratio is defined in previous studies as the ratio of the effective energy (or effective temperature) of the system to the thermal energy^28,29^. Using the assumption by Mizuno *et al*.^20^ that the magnitudes of the equilibrium and nonequilibrium fluctuation energies are additive, we can determine the chemically produced, active fluctuating force spectrum^20^ $\langle {f}_{nonequ.}^{2}\rangle$ by subtracting the thermal-equilibrium spectrum (*C*_*equ*._; estimated by use of AMR) from the total fluctuation spectrum (*C*_*total*_; measured by PMR). In addition, we also assume that time-averaged local environmental stiffnesses, <k*>, remains constant over time. We examine this additional assumption in the next section. Under both assumptions, we disentangle the equilibrium and non-equilibrium force spectra, even though our measurements of active and passive microrheology are not made time-synchronously.

1 $$\langle \,{f}_{nonequ.}^{2}\rangle ={\langle {k}^{\ast }\rangle }^{2}({C}_{total}-{C}_{equ.})={\langle {k}^{\ast }\rangle }^{2}\left({C}_{total},-,\frac{2{k}_{B}T}{{k}^{{\rm{{\prime} }}{\rm{{\prime} }}}\omega }\right)$$

We find that at frequencies lower than about 1 Hz the fluctuations measured by PMR (*C*_*total*_) have a magnitude larger than the expected equilibrium fluctuations based on AMR (*C*_*equ*._) for cells cultured on the stiffer substrate^27^ (Fig. 1D). At frequencies higher than 10 Hz the PMR and AMR results coincide, showing that at high frequencies the system is in equilibrium, with only equilibrium forces acting on the probe particle. Our measurement of the comparison between PMR and AMR is consistent with previous studies^21,32^. Here, we find that stronger nonequilibrium fluctuating forces, $\langle \,{f}_{nonequ.}^{2}\rangle$, are dependent on cells cultured on stiffer substrates (Fig. 1E) and the nonequilibrium fluctuating forces at 1 Hz increase with substrate rigidity (Fig. 1F). This result indicates that cells can generate stronger nonequilibrium fluctuating forces, which might be regulated by molecular-motor dynamics, to sense the mechanics of their microenvironments.

### The correlation between intracellular nonequilibrium fluctuating stresses and local stochastic intracellular stiffness modulus

**Figure 2:**
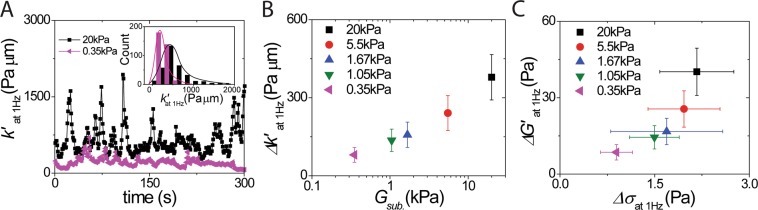
Correlation between fluctuations in intracellular stiffness modulus and nonequilibrium stress. (**A**) Fluctuation of intracellular stiffness (*k*′) at 1 Hz as a function of time via using active microrheology. Insert shows the probability histogram of *k*′ _at 1Hz_ overtime period shows the positive-skew distribution. The mean of *k*′ is 14%, and 3% greater than the median of *k*′, when cells attached to the stiffer (*i.e*., 20 kPa) and the softer (*i.e*., 0.35 Pa) substrates, respectively. (**B**) The result of fluctuations of intracellular stiffness *Δk*′ increase for cells cultured on stiffer substrates. *G*_*sub*,_ is the elastic modulus of the substrate. (**C**) Magnitudes of fluctuations in intracellular stiffness modulus (*ΔG*′) increase with magnitudes of fluctuations in intracellular nonequilibrium stress (*Δσ*). Error bars are standard deviation of the mean from ten independent measurements from individual probed particles in each cell.

To understand how living cells adjust their mechanical responses to the active, nonequilibrium fluctuating forces, we use oscillatory optical tweezers and phase lock-in detection method to measure intracellular stiffness (*k*′). Then, we determine the variance of *k*′ (*i.e*., the second moment or the standard deviation of *k*′ at 1 Hz over a 300-second duration as shown in Fig. 2A) and the probability histogram of *k*′ over the period, as shown in the insert of Fig. 2A. To examine if <*k*′> remains constant over time, we calculate the mean of *k*′ at 1Hz from the different measuring time duration. When a cell was cultured on the stiffer substrate, <*k*′>_at 1Hz_, is 633 Pa μm and 641 Pa μm averaged from 0~150 s and 150~300 s, respectively (as shown in Fig. 2A). It shows that the “time-averaged” local environmental stiffnesses have a 1% variation from the different measuring time durations. Our data support our assumption that the “time-averaged” local environmental stiffness spectrum <k*> remains constant.

We find larger fluctuations of intracellular stiffness (*Δk*′) for cells cultured on substrates with larger elastic moduli (Fig. 2B). We define the fluctuating stiffness modulus (*ΔG*′) through a generalization of the Stokes relation (*ΔG*′=*Δk*′/6*π a*)^10,20,29^ and the nonequilibrium fluctuating stress $\Delta \sigma ={\langle {f}_{nonequ.}^{2}\rangle }_{at\,1Hz}^{0.5}/(\pi {a}^{2})$ from the nonequilibrium fluctuating force at 1 Hz (Fig. 1F), where “*a*” is the radius of the probe particle. Here, we find that as cell cultured on stiffer substrates, an intracellular nonequilibrium fluctuating stress increase with the increasing fluctuations of intracellular stiffness modulus^27^ (Fig. 2C).

### Response of intracellular stiffness modulus to intracellular stress is nonlinear

**Figure 3:**
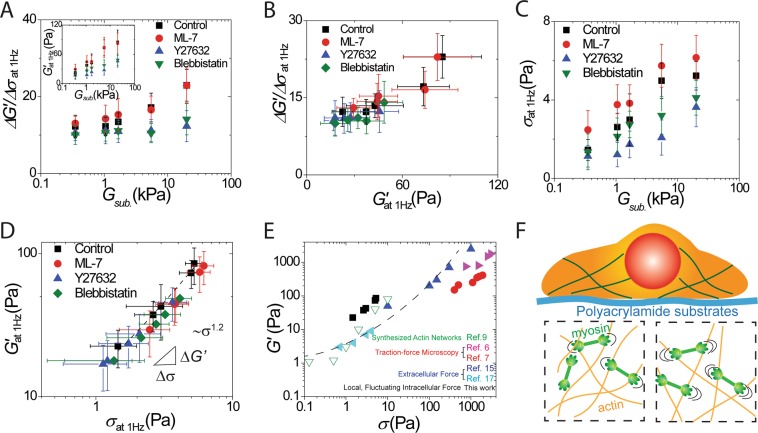
Nonlinear response of intracellular stiffness modulus to intracellular stress. (**A**) Experimental results of the ratio of the fluctuating stiffness modulus to the nonequilibrium fluctuating stress (*ΔG*′*/Δσ*) and (see insert) intracellular stiffness modulus (*G*′) when cells are cultured on substrates of various rigidity. *G*_*sub*._ is the elastic modulus of the substrate. (**B**) The ratio of the fluctuating stiffness modulus to the nonequilibrium fluctuating stress (*ΔG*′*/Δσ*) as a function of intracellular stiffness modulus. (**C**) Intracellular stress (*σ*) as a function of substrate rigidity (*G*_*sub*._). (**D**) Intracellular stiffness modulus as a function of intracellular stress when cells are cultured on substrates of different stiffness. The mechanical response of intracellular stiffness modulus is a non-linearly increasing function of intracellular stress, with power 1.2. Error bars are standard deviation of the mean from ten independent measurements from individual probed particles in each cell. (**E**) The stiffness moduli of different cell types and flexibly cross-linked actin networks versus either extracellular stress or cellular-traction stress. The dashed curve is qualitative. (**F**) Schematic illustrating the myosin-regulated nonlinear-mechanics system in a living cell where the nucleus is shown as a red blob surrounded by cytoskeleton proteins. Motor proteins that exert forces in actin network are shown in the lower panels.

To study how the relationship between intracellular stiffness modulus and intracellular stress (*σ*) varies with microenvironment rigidity, we determine the intracellular stress via variations of the fluctuations in stiffness modulus and nonequilibrium stress in living cells. The ratio of the fluctuating intracellular stiffness modulus to the intracellular nonequilibrium fluctuating stress (*ΔG*′*/Δσ*) is not zero and increases with the substrate rigidity (Fig. 3A). The ratio of *ΔG*′ to *Δσ* also increases with intracellular average stiffness modulus, indicating stress-depended stiffness nonlinear mechanical behavior (Fig. 3B). To determine the intracellular stress, we integrate the ratio of the nonequilibrium fluctuating stress (*Δσ*) to the fluctuations of intracellular stiffness (*ΔG*′) over all values of intracellular stiffness modulus (*G*′) (*∫*(*Δσ/ΔG*′) *dG*′)^27^. Here, the value for the linear stiffness modulus *G*_0_′ in the absence of intracellular stress is determined to be 5 Pa^15^, which is also in the range of unstressed cross-linked actin networks^33^.

We find that the intracellular stress (*σ*) of cells attached to a stiff substrate is significantly larger than those attached to a soft substrate (Fig. 3C). Our results also show an increasing intracellular stiffness modulus as a function of increasing intracellular stress (Fig. 3D). This shows a non-linearity of the dependence of cell rigidity on cell stress, with a power 1.2 ± 0.02 (black dash line in Fig. 3D)^27^, which is similar to that of active networks with exponent 1.3^12^ and cellular cortex with exponent 1.13^14,15^. To compare with previous results, all of the results show a similar nonlinear mechanical behavior (Fig. 3E) with a strong non-linearity of stiffness modulus for flexibly cross-linked actin networks versus extra-stress^12^ and for different cell types versus cellular-traction stress^6,7^, or extracellular stress^15,17^. Instead of application of an external stress, our results indicate that cytoskeletal networks can be turned into a contractile material in living cells by motor activity. The data show that cells and cytoskeletal polymers are a stress-stiffening material in which the network stress controls the stiffness. This indicates that the stiffness of adherent cells would increase with intracellular stress and contractile tension.

To investigate how intracellular stress regulates cell mechanical properties, we vary intracellular stress with drugs (*i.e*., ML-7, Y-27632, and blebbistatin) that alter motor proteins. Cells treated with Y-27632 and blebbistatin exhibit a decrease in time-averaged intracellular stiffness modulus and intracellular stress (the insert of Fig. 3A,C). There is no significant difference in intracellular stress and time-averaged intracellular stiffness modulus for cells treated with ML-7. Treatments with Y-27632 and blebbistatin inhibit the cell’s response to substrate rigidity^27^ (Fig. 3D), allowing the intracellular stresses to mimic the mechanical properties of the microenvironment. Our measurements of intracellular stress in response to substrate rigidity have a similar trend to those of previous measurements by traction-force microscopy using either fluorescent particles embedded in a substrate^7^ or micropillars^34^ to observe substrate deformations. With traction-force microscopy, pulling or contraction by cells would be measured as a time-averaged cellular stress that balances the traction stresses exerted on the substrate by the cells. The results show that fibroblasts tend to match their internal stiffness to that of their substrates up to 20 kPa. Also actin remodeling in cells is enhanced with increasing substrate rigidity^35^, suggesting that actin stress fibers may act as force sensors that transmit tension to focal adhesion complexes, possibly via contribution from myosin motors. Our results are qualitatively consistent with previous studies that show cellular traction stress is mainly regulated by ROCK, but not by myosin light chain kinase^36–40^.

## Discussion and Conclusions

We report the noise spectra in a nonequilibrium thermodynamic system of the nonlinear mechanical cytoskeleton network in a living cell. We use microrheology to study intracellular stress in response to substrate rigidity (Fig. 3F). We further vary intracellular stress using drugs that inhibit motor activity and produce a single master curve with a power-law dependence. Results of this study, in particular the data shown in Fig. 3D, describe intracellular stiffness modulus as a strongly nonlinear function of intracellular stress. This suggests that the motors induce internal stress and tension that produce a nonequilibrium and nonlinear state. Our finding emphasizes the close analogy of motor-driven internal stress with external shear stress. These aspects of intracellular stress and stiffness modulus give at least one explanation of the mechanical noise inside cells. Further probing of the different sources of noise in living cells may reveal other mechanical responses of the dynamic cytoskeleton network.

We show that fluctuations of a nonequilibrium thermodynamic system provide a direct means to characterize the nonlinear mechanical properties of intracellular stiffness modulus as a function of intracellular stress, which have been difficult to obtain by other approaches. Our data provide evidence that cells can modulate their mechanical properties by modulating their inner mechanical stress. It opens the question as to how living systems use these fluctuations as an energy-efficient mechanism to adapt to their microenvironment. Thus, further examination of these fluctuations will advance the understanding of how cells sense and respond to their mechanical environment, leading to new designs in biomaterials and new therapies for diseases linked to cellular mechano-transduction.

## Materials and Methods

### Preparation of HeLa cells and polyacrylamide thick films

HeLa cells are cultured in Dulbecco’s modified eagle medium supplemented with high glucose (Gibco #11965092), 10% fetal bovine serum (Gibco #16140071), 1% penicillin/streptomycin (Invitrogen #15140-122), 7.5% sodium bicarbonate, 200 mM glutamine, and 1% G418 solution (Thermo Fisher #10131027). The cell line is generously provided by Dr. Keiju Kamijo at the National Institutes of Health. Cells are seeded onto polyacrylamide (PA) substrates coated with sulfo-SANPAH cross-linker and collagen type I (0.2 mg/ml) on 22 **×** 22 mm cover-slips. Different PA substrates, having varying elastic moduli, are prepared^41,42^. Cells are grown under standard culture conditions (37 °C, 5% CO_2_, humidified environment). To explore the internal cell mechanics with respect to the activity of intracellular motors, we treat myosin-inhibitors for 1 hour using (1) ML-7 (20 μM; Sigma-Aldrich #I2764), which is a potent and selective inhibitor of myosin light chain kinase; (2) Y-27632 (10 μM; Sigma-Aldrich #Y0503), which inhibits the Rho-associated protein kinase (ROCK) and thus inhibits ROCK-mediated myosin light chain phosphorylation; and (3) blebbistatin (20 μM; Sigma-Aldrich #B0560), which binds to the myosin ATPase and slows phosphate release.

### Active micro rheology with oscillatory optical tweezers

We construct our oscillatory optical tweezer system with an IR laser (30 mW, wavelength = 1064 nm, Spectra-Physics). The laser is highly focused by an oil-immersion microscope objective lens (Olympus, PlanFluo, 100X, numerical aperture = 1.3) to trap a 1 μm polystyrene particle (Thermo Fisher Scientific #4009A). A schematic diagram of the experimental setup is shown in our previous published papers^43–45^. Movements of the trapped particle, tracked by the other IR laser beam (0.5 mW, wavelength = 980 nm, Thorlabs, Inc.), are detected by a quadrant photodiode (QPD, Hamamatsu #S7479). The voltage reading of the QPD is maintained to be within the linear range of the particle displacement from the trapping center^27,45^. A lock-in amplifier (Stanford Research #SR830) referenced to a sinusoidal signal that drives the PZT-driven mirror (Physik Instrumente #S-224). This provides great sensitivity to determining the displacement amplitude and phase shift of the trapped particle. The cell culture chamber containing an objective heater (Bioptechs) is mounted on an inverted microscope (Olympus IX81). Combining optical tweezers with known optical spring constants as force sensors and phase-sensitive detection^24,45–47^, we measure the viscoelasticity in living cells. Notably, since the cytoplasm has different optical properties from that of the medium, the optical spring constant used for measuring intracellular stiffness modulus would be corrected via the refractive index mismatching^24^. The rescaling reduced the optical spring constant of the value determined from a particle in water. This assumption might lead to a 10% error due to the optical properties of heterogeneous media in living cells.

### Passive microrheology

To measure the total fluctuation spectrum using passive microrheology, the fluctuations of a 1 μm polystyrene particle (Thermo Fisher Scientific #4009A) entrapped in HeLa cells are recorded by a fast camera. The fluctuations of the particle position, which is the absence of optical tweezers force, is recorded for 300 seconds with signal acquisition at 500 frames/sec. To combine active and passive microrheology, we compare the results of total fluctuation and the thermal-equilibrium fluctuation from the same probed particle. Then, average the ten independence measurements from individual probed particles in each cell. Note, the measurements of active and passive microrheology are at the same site but are not time-synchronous.

### Data analysis

**Figure 4:**
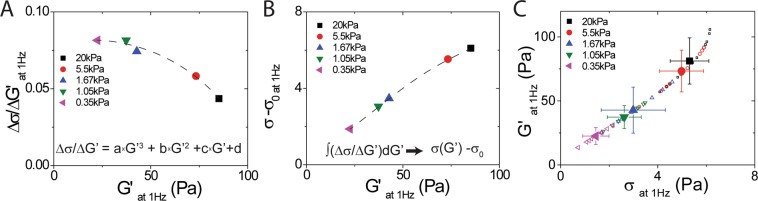
Data analysis of intracellular stress. (**A**) Polynomial fitting of *∆σ/∆G*′ as a function of intracellular stiffness, *G*′ and (**B**) relative intracellular stress, *σ - σ*_0_, as a function of intracellular stiffness calculated by integrating the third polynomial function, *∆σ/∆G*′ (*G*′). (**C**) The intracellular stiffness as a function of intracellular stress, calculated from integrating the polynomial function, when cells were cultured on different rigidity substrates.

To determine intracellular stress (*σ*), we calculate the ratio between *Δσ* and *ΔG*′ which is the fluctuation of intracellular stress and the fluctuations of intracellular stiffness, respectively. Both of *Δσ* and *ΔG*′ are averaged independent cells cultured on different rigidity substrates. Each averaged value is calculated from ten independent measurements from individual probed particles in each cell. Here, we integrate the* Δσ/ΔG*′ over all values of intracellular differential stiffness (*∫*(*Δσ/ΔG*′)*dG*′). First, we use a third-order polynomial form to fit the *Δσ/Δ G*′ as a function of intracellular stiffness (*G*′), as shown in Fig. 4A. Then, the relative intracellular stress (*σ − σ*_0_), as shown in Fig. 4B, is calculated by integrating the polynomial function, *Δσ/ΔG*′ (*G*′). *σ*_0_ is the value independent of intracellular stiffness, *G*′, for the integrating polynomial function. Since *G*′ is never zero at any *σ*, we are looking for the value for the linear modulus *G*_0_′ in the absence of intracellular stress, *σ* = 0. Here, *σ*_0_ is calculated from the value for the linear stiffness modulus *G*_0_′ in the absence of intracellular stress is determined to be 5 Pa, which is also in the range of unstressed cross-linked actin networks. The stress-dependent stiffness, calculated by integrating the polynomial function, as cells culture on different rigidity substrates shows in Fig. 4C. Using the same protocol, we determine intracellular stress-dependent stiffness for each drug treatment, including ML-7, Y-27632, and blebbistatin, as shown in Fig. 3D.
